# Using population data for assessing next-generation sequencing performance

**DOI:** 10.1093/bioinformatics/btu606

**Published:** 2014-09-17

**Authors:** Darren T. Houniet, Thahira J. Rahman, Saeed Al Turki, Matthew E. Hurles, Yaobo Xu, Judith Goodship, Bernard Keavney, Mauro Santibanez Koref

**Affiliations:** ^1^Oxford Gene Technology, Begbroke Science Park, Oxford, Oxfordshire, OX5 1PF, ^2^Institute of Genetic Medicine, Newcastle University, International Centre for Life, Central Parkway NE1 3BZ, Newcastle upon Tyne and ^3^The Wellcome Trust Sanger Institute, Wellcome Trust Genome Campus, Hinxton, Cambridge, CB10 1SA, UK

## Abstract

**Motivation**: During the past 4 years, whole-exome sequencing has become a standard tool for finding rare variants causing Mendelian disorders. In that time, there has also been a proliferation of both sequencing platforms and approaches to analyse their output. This requires approaches to assess the performance of different methods. Traditionally, criteria such as comparison with microarray data or a number of known polymorphic sites have been used. Here we expand such approaches, developing a maximum likelihood framework and using it to estimate the sensitivity and specificity of whole-exome sequencing data.

**Results**: Using whole-exome sequencing data for a panel of 19 individuals, we show that estimated sensitivity and specificity are similar to those calculated using microarray data as a reference. We explore the effect of frequency misspecification arising from using an inappropriately selected population and find that, although the estimates are affected, the rankings across procedures remain the same.

**Availability and implementation**: An implementation using Perl and R can be found at busso.ncl.ac.uk (Username: igm101; Password: Z1z1nts).

**Contact**: Darren.Houniet@ogt.com; mauro.santibanez-koref@newcastle.ac.uk

## 1 INTRODUCTION

The identification of sequence variants predisposing to diseases that follow Mendelian inheritance patterns is a common application of next-generation sequencing technologies (Ng *et al.*, 2010a, b, c; Wang *et al.*, 2010). A key step in these approaches is the identification of variants shared among affected relatives (Ng *et al.*, 2010a, b, c). Usually a variant is defined as a deviation from a reference sequence. Next-generation sequencing of material enriched for exonic sequences has been successful in many cases, but has failed to identify the causative variants in others (Bamshad *et al.*, 2011; Gilissen *et al.*, 2011). Such apparent failures may have many causes but also focus attention on the desirability of simple measures to assess the results of the sequencing and analysis pipelines used. In disease-mapping efforts, it is clearly desirable to have a high probability of identifying a true variant while the number of falsely identified variants remains low. This will reduce the amount of work needed for validation of candidate variants, which is typically undertaken using Sanger sequencing or genotyping approaches.

Our objective is to develop a simple and flexible approach for assessing the performance of a whole-exome or -genome sequencing experiment. It should allow assessing the whole process, from sample preparation to variant calling. The focus is on the detection of single-base sequence variants as opposed to changes in copy number or large rearrangements. One approach is to compare the identified variants with variants known to be present or absent. For this purpose, variants are commonly compared with the results of genotyping microarrays (e.g. Ng *et al.*, 2009). This allows for the probability of a specific variant at a given position in an individual to be considered as either 0 or 1. The price of this certainty is additional experimental costs.

An alternative is to use variants where the probability of occurrence in a specific sample can be ascertained, thus allowing the probability of a variant present at a specific position to assume values other than 0 or 1. Here we formalize the second approach using sites known to be polymorphic in the human population. This approach can be seen as an extension of methods that rely on quality criteria such as the number of variants found in sites known to be polymorphic in the human population (e.g. Challis *et al.*, 2012; Marth *et al.*, 2011). We compare our results with those obtained using microarray data and use our method to assess different analysis pipelines and to explore the importance of analysis parameters and coverage depth. As we are using known polymorphisms, we also investigate the influence of the population that was used to characterize the polymorphisms on the results.

The use of microarrays to assess the accuracy of variant identification is likely to lead to biased results, as the polymorphisms present on microarrays usually exclude variants such as small insertion/deletions (indels). Such variants also represent a challenge for variant calling in next-generation sequencing. In the last paragraph, we therefore explore different analysis pipelines for indel calling.

The results of such comparisons can be summarized in many ways. Commonly used metrics include, for example, the number or proportion of previously reported variants among the detected deviations from the reference sequence. As our focus is on a dichotomous outcome, i.e. the presence or absence of a variant at a particular position, we use here the probabilities of identifying the variant at a site that carries a variant and of identifying no variant at a site where only the reference sequence is present. We refer to these probabilities as sensitivity and specificity.

## 2 METHODS

*Parameter estimation*: We designate with *M* the presence of a variant allele and with *D* the detection of a variant allele. Correspondingly, $\bar{M}$ and $\bar{D}$ represent the absence of a mutant allele and the non-detection of a mutant allele. For an autosomal locus we have:
(1)
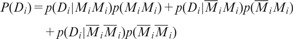

(2)
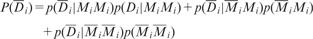



Assuming Hardy–Weinberg equilibrium, we obtain for the genotype frequencies $p(M_{i}M_{i})=f^{2},p(M_{i}{\bar{M}}_{i})=2f_{i}91-f_{i}$ and $p({\bar{M}}_{i}{\bar{M}}_{i})={\left(1-f_{i}\right)}^{2}$ where *f_i_* designates the frequency of the variant allele. We further designate with *s* the sensitivity s=p(D|M) and with *u* the specificity $u=p(\bar{D}|\bar{M})$ and obtain for the remaining terms: $p(D_{i}|M_{i}M_{i})=s(2-s)p(D_{i}|M_{i}{\bar{M}}_{i})=s+(1-s)(1-u),p(D_{i}|{\bar{M}}_{i}{\bar{M}}_{i})=1-u^{2},p({\bar{D}}_{i}|M_{i}M_{i})={\left(1-s\right)}^{2},p({\bar{D}}_{i}|M_{i}{\bar{M}}_{i})=(1-s)u$ and $p({\bar{D}}_{i}|{\bar{M}}_{i}{\bar{M}}_{i})=u^{2}$.

We treat all sites as independent and assume that the detection probability for one site is independent from that for another, and thus, for an individual, the likelihood is $l(s,u)=\prod_{i\epsilon S_{D}}P(D_{i})\prod_{j\epsilon S_{\bar{D}}}P({\bar{D}}_{j})$, where *S_D_* represents the set of sites where a variant was detected and $S_{\bar{D}}$ represents the sites where only the reference was observed. The estimates of *s* and *u* are the values that maximize this likelihood. Owing to the linkage disequilibrium, we cannot assume that the occurrence of variants at neighbouring sites is independent. Therefore, we perform the calculation by choosing random sets of sites where the sites in the same set are either separated by at least 500 kb or on different chromosomes. Here we perform the calculations over 1000 of such sets. This leads to a series of estimates for *s* and *u*, allowing us to compute the median of these estimates.

When several individuals are analysed and we could assume that their genotypes are independent, the likelihood for a group of *K* individuals can be described as $l(s,u)=\prod_{k=1}^{K}l_{k}(s,u)$.

Here we analyse each individual separately and report the median, as well as the 95% confidence interval, determined by bootstrapping.

*Sequence data*: Targeted whole-exome sequencing was carried out for 31 (12 + 19) samples using an Illumina Genome Analyser IIx. Agilent 38 Mb target (hg18) positions were obtained from Agilent, and the sequences corresponding to these positions were obtained using the Galaxy website. Build 36.1 of the human genome (hg18) was used as a reference sequence (https://genome.ucsc.edu/).

*Genotyping arrays*: Genotype chip data were available for 19 of the 31 samples, composing of 557 124 SNPs on the Illumina 660 genotype chip. In all, 10 762 of these SNPs were located within the target regions.

*Comparison of array and sequencing data*: As described in Section 1, our analysis focuses on the ability to detect variants, and therefore, we assess at any position whether a variant was detected. The sensitivity is defined as the number of sites in which both sequencing and microarrays detected a deviation from the reference sequence divided by the number of sites where a variant was detected by using the microarrays. Correspondingly, the number of sites where both methods detected no deviation from the reference sequence divided by the number of sites where the microarray detected only the reference residue was used as an estimate of the specificity.

*Selection of polymorphisms*: The HapMap database contained 4 083 713 SNPs (CEU population, build 36, downloaded October 28, 2010). Of these, 10 165 overlapped with the on-target genotype chip SNPs. Allele frequency data were obtained for each of these SNPs from the HapMap database. All 10 165 available SNPs were used in the analysis.

*Sequence analysis*: Unless specified otherwise, reads were aligned to the whole human genome (hg18) using the following aligners: Bowtie 0.12.8 (Langmead *et al.*, 2009), BWA 0.6.2 (Li and Durbin, 2009), GSNAP 20120720 (Wu and Nacu, 2010), NovoAlign 2.07.13 (http://www.novocraft.com), SOAP2 2.21 (Li *et al.*, 2009b) and SSAHA2 2.5.5 (Ning *et al.*, 2001). Variants were identified using either Varscan 2.3.1 (Koboldt *et al.*, 2009) or Samtools 0.1.8 (Li *et al.*, 2009a). Unless specified otherwise, the default parameters were used for each program. Coverage was assessed from the pileup files. Coverage depth was varied by sampling with replacement from the SAM files.

*Indel calling*: Twelve of the samples were used for the indel analysis. Reads were aligned to the human genome reference (hg19) sequence using Bowtie2 (Langmead and Salzberg, 2012), BWA and NovoAlign. Indels were identified using Samtools 0.1.8, Dindel 1.01 and GATK 2.6.4 (DePristo *et al.*, 2011; McKenna *et al.*, 2010). For Dindel, a minimum variant coverage of seven was used, and for all other programs, the default parameters were used.

*Other metrics*: There is a large number of metrics that can be used to assess the quality of the sequencing experiments. Here we will only explore the fraction of target bases covered at least once, average coverage depth and the fraction of target bases covered at least 10-fold.

## 3 RESULTS

In this section, we first explore some applications of the method proposed, then compare its results with estimates generated using the genotypes obtained through the microarray as the true genotypes. The method only requires that the allele frequencies in the population from which the samples were drawn are known. However, this may not be the case; therefore, we explore the effects of population misspecification.

**Fig. 1.**
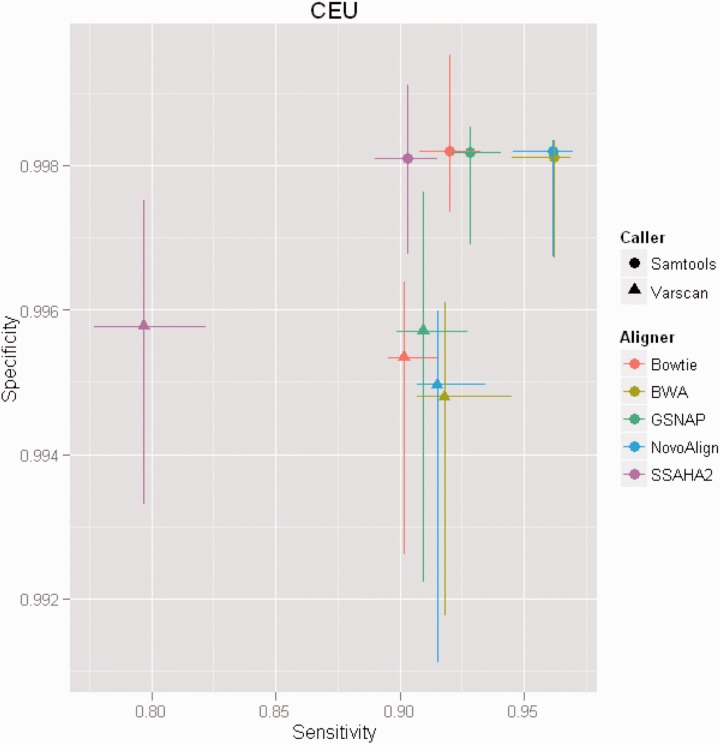
Comparison of estimated specificity and sensitivity for different pipelines

*Pipelines*: Figure 1 compares sensitivity and specificity estimates achieved using different alignment and variant calling program combinations. The values are based on 31 samples. For eight samples, SSAHA2 failed to produce results and generated the messages error: memory allocation failed cannot allocate memory or error: “memory allocation for array of fasta structures failed cannot allocate memory”. Represented for each combination are the median and the upper and lower quartiles. Interestingly, the alignment programs appear to have a stronger effect on sensitivity, whereas the variant calling programs appeared to effect specificity more strongly. All aligners yield similar specificities when used in combination with Samtools, and the combinations NovoAlign/Samtools and BWA/Samtools provided the highest sensitivity. Therefore, we used NovoAlign as aligner and Samtools as variant caller as the standard pipeline for subsequent analyses. The estimates for the specificity using Samtools with any aligner were in excess of 0.998.

**Fig. 2.**
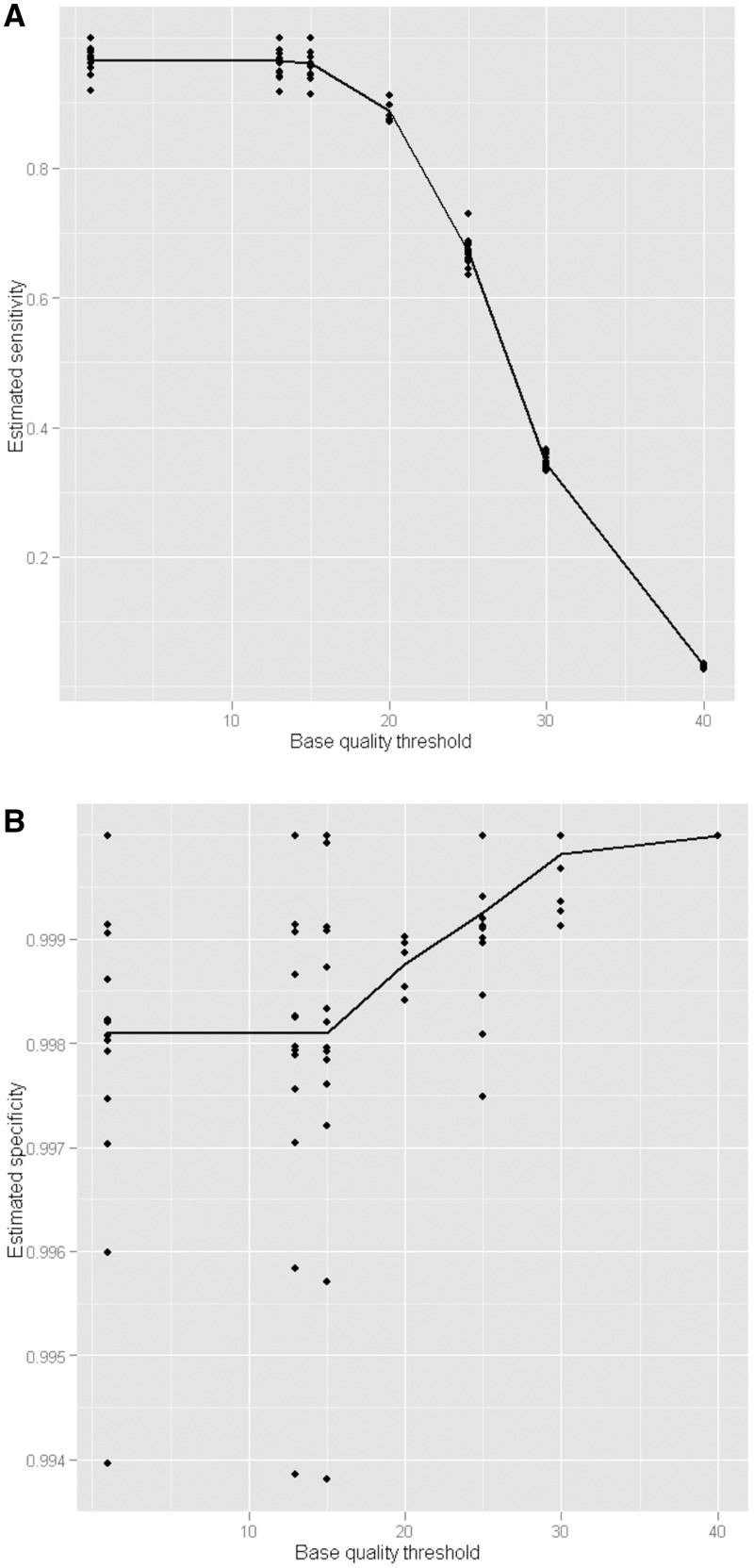
Exploring the effect of parameter choice. Represented is the effect of minimum base quality threshold on estimated sensitivity (Panel A) and specificity (Panel B). Each point on the graph represents the result for a single sample analysed using the minimum base quality threshold given in the abscissa

*Parameter selection*: Each alignment and variant calling program has a range of parameters, which can be set by the user. In general default, values are provided, but these may not always be appropriate, and altering these parameters can have a marked effect on sensitivity and specificity. Our procedure can be used to investigate such effects. As an example, we explore the effect of varying the quality thresholds for variant calling using the NovoAlign/Samtools pipeline (Fig. 2). This threshold is the minimum base quality at a position, for a read, required for that read to be included in the variant call for that position. Figure 2A shows that altering this parameter has a dramatic effect on sensitivity, with a rapid drop when the values are set above 20. The effect on specificity (Fig. 2B) is more modest, and increasing the values beyond 30 has only a limited effect.

**Fig. 3.**
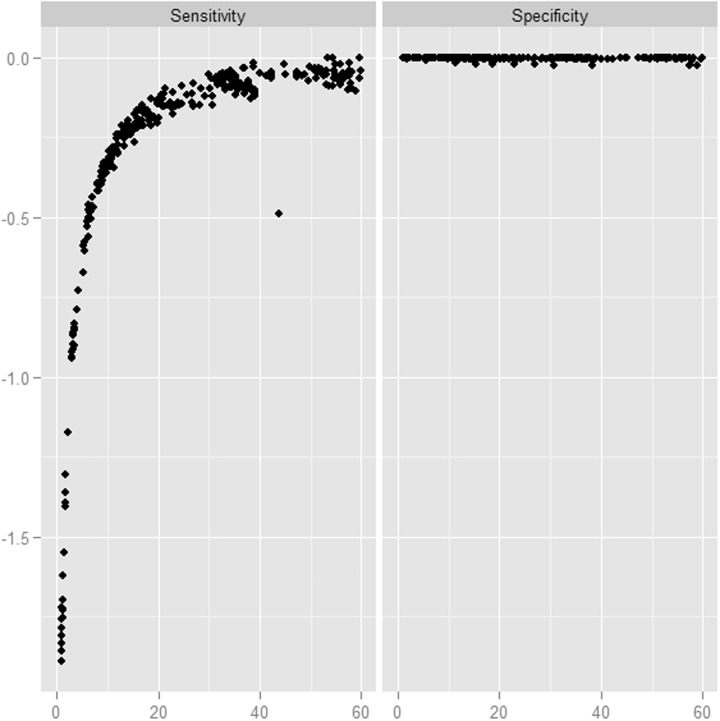
Effect of average coverage. Each point represents the sensitivity (log scaled) achieved for a sample at a given value

*Coverage*: Figure 3 explores the effects of average coverage. As expected, sensitivity increases with increasing coverage. With the parameters we used, there is a substantial loss of sensitivity when the average coverage was <40-fold. Specificity shows little variation with increasing depth. At low coverage, finding evidence for a variant generally becomes more difficult, thus leading to a low sensitivity and high specificity.

**Fig. 4.**
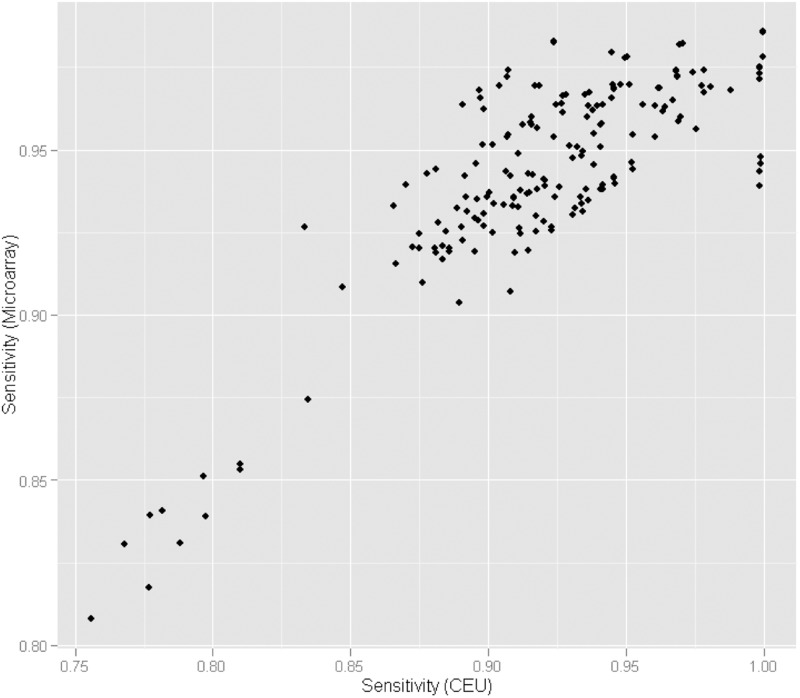
Correlation between sensitivity estimates from microarray data and using CEU population frequencies. Each point represents the sensitivity value obtained for individuals and analysis pipelines

*Microarray comparison*: For 19 samples, data were available from both platforms. The variants were called using the NovoAlign/Samtools pipeline with default parameters. Table 1 compares sensitivity and specificity figures estimated using genotype microarray data (fourth column) and our method with two different sets of allele frequencies (second and third column). The frequencies used for the estimates in the third column were calculated from the genotyping results for the 19 samples. We see that compared with the microarrays, both specificity and sensitivity estimates are slightly lower by the frequency method using the CEU population frequencies (second column). One possible contributor to this effect is that the estimates are distorted because the allele frequencies do not match the frequencies in our sample. In fact, the difference is smaller when the allele frequencies used are derived from the genotyping results (third column). It should also be mentioned that specificity estimates are in the order of 0.999. These estimates, however, are based only on a limited number of polymorphic sites (10 165 sites), suggesting that the ability to adequately assess changes in the specificity will be limited. This is reflected in the correlation between the estimates obtained from microarrays and from population frequencies. Although there is a good correlation between the estimates for the sensitivity (Fig. 4; $R^{2}=0.71,P=4x10^{-50}$), the correlation for specificity is rather poor although still significant ($R^{2}=0.39,P=7x10^{-21}$).

**Table 1.** Mean sensitivity and specificity estimates

Sensitivity and Specificity	Estimated from		
	CEU frequencies^a^	Sample frequencies^b^	Microarray^c^
Sensitivity	0.962	0.979	0.984
(95% CI^d^)	(0.945–0.970)	(0.962–0.986)	(0.982–0.986)
Specificity	0.998	0.999	0.999
(95% CI)	(0.997–0.998)	(0.999–0.999)	(0.999–1.00)

*Notes*: Represented are the estimates for the specificity and sensitivity of the NovoAlign/Samtools pipeline.

^a^Allele frequencies for the Hapmap CEU population.

^b^Allele frequencies calculated from the genotyping genotyping results for the 19 samples.

^c^Genotypes determined using the Illumina 660 W chip.

^d^95% confidence interval for the mean, determined by resampling.

**Fig. 5.**
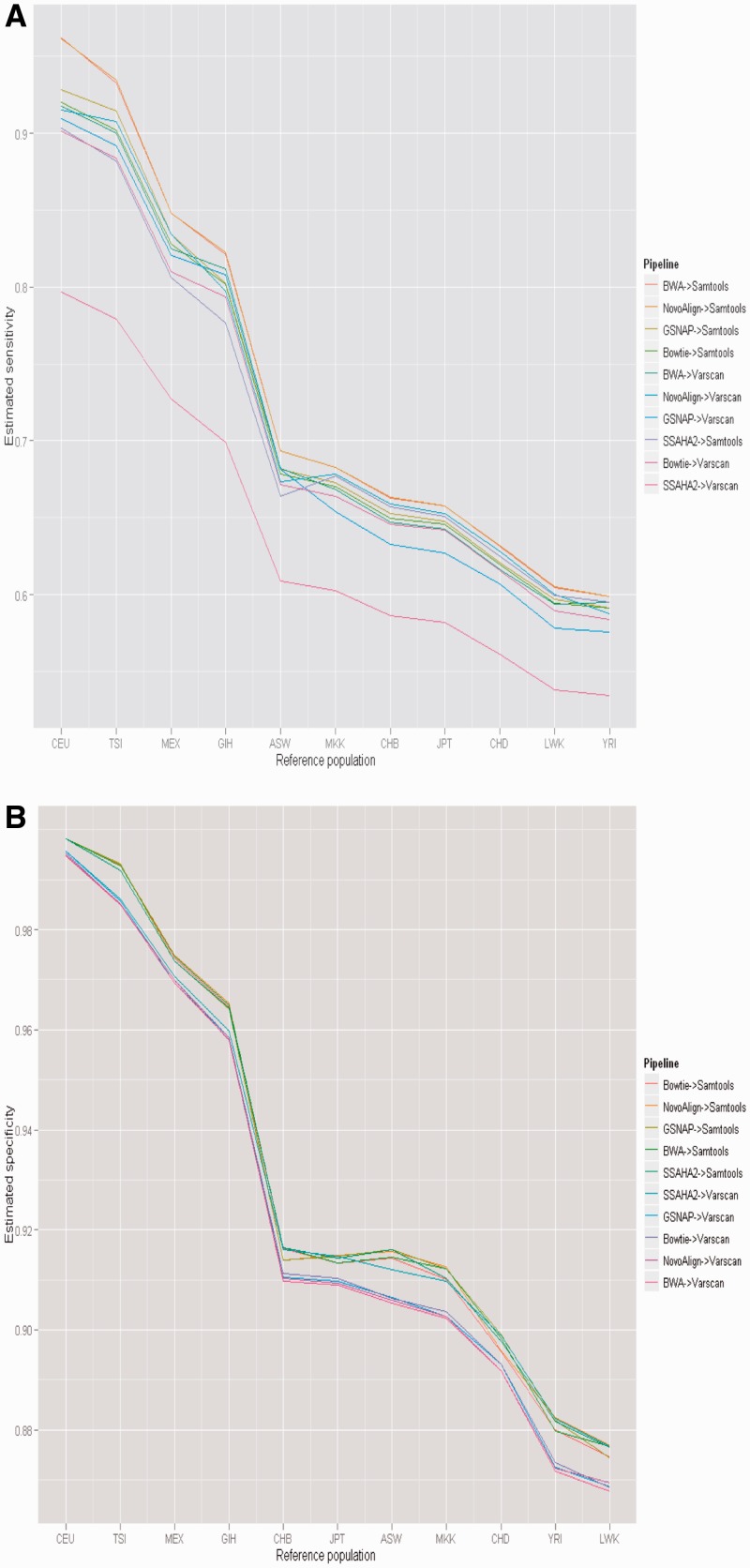
Effect of reference population misspecification. Values represent the average sensitivity across all individuals in our study. CEU: Utah residents with northern and western European ancestry from the CEPH collection; TSI: Tuscan in Italy; MEX: Mexican ancestry in Los Angeles, California; GIH: Gujarati Indians in Houston, Texas; ASW: African ancestry in southwest USA; MKK: Maasai in Kinyawa, Kenya; CHB: Han Chinese in Beijing, China; JPT: Japanese in Tokyo, Japan; CHD: Chinese in Metropolitan Denver, Colorado; LWK: Luhya in Webuye, Kenya; YRI: Yoruban in Ibadan, Nigeria

*Allele frequency effects*: The method we propose uses allele frequencies in the estimation of sensitivity and specificity. Allele frequencies vary between populations, and the ethnicity of the individuals who provided a sample may not always be precisely known. We therefore explored how the use of alternative allele frequency data from a variety of populations would influence our results. Figure 5 explores the effects of using the allele frequencies for 11 HapMap populations in the estimation of the performance of the pipelines used previously. The figure suggests that misspecification of population frequencies tends to lead to lower estimates of both sensitivity and specificity. However, the lines connecting the values for different pipelines calculated using different allele frequencies tend to be parallel. This indicates that the results are correlated (*P* < 0.01 for all comparisons), suggesting that although the estimates may vary, the different pipelines will tend to be in the same order.

**Fig. 6.**
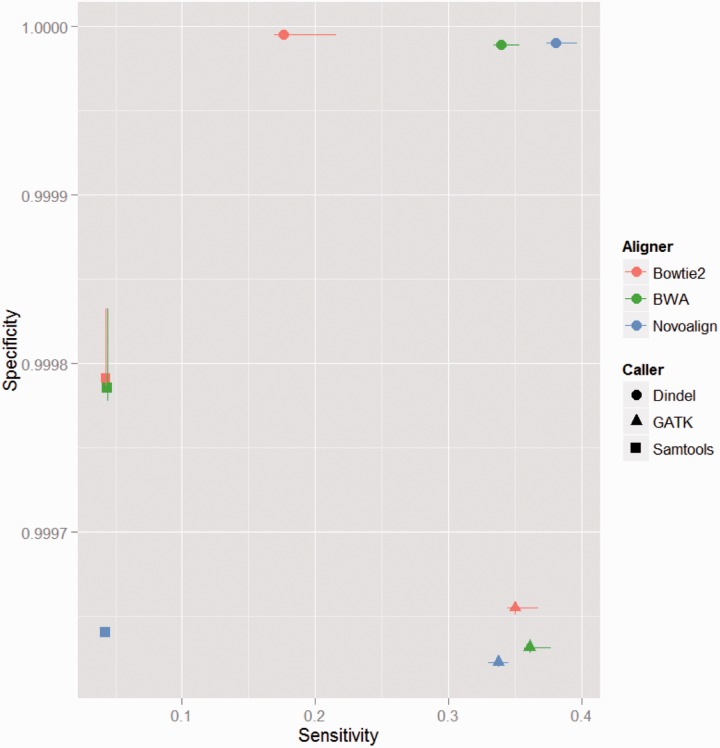
Sensitivity of different indel detection pipelines

*Indel calling*: This analysis is based on 12 samples and 8365 indel variants included in the 1000 genomes data (CEU). The low number of positions considered makes estimation of specificity problematic. One possibility is to include in the analysis sites where no indel has been reported in the databases and assign them a low probability to carry a variant. Here we followed this path assuming that the frequency of a rare allele in a position where no variant has been described to be 10^−^^8^. Figure 6 shows the results for different indel calling pipelines. For all pipelines, the sensitivity is substantially lower than the values obtained for single-base substitutions (compare with Fig. 1). We also observe that Samtools has the lowest sensitivity, whereas GATK performed best. We also saw that the combination BWA/GTAK performed best. The higher specificity value achieved by Dindel is likely due to the minimum variant call threshold used in analysis. The average estimates for sensitivity using the bowtie/GATK and NovoAlign/GATK pipelines are 0.35 and 0.34, respectively.

## 4 DISCUSSION

The approach we presented here estimates two parameters, the sensitivity and the specificity of variant calls in a next-generation sequencing experiment, by comparing the observed variants with population allele frequency data in a maximum likelihood framework. We illustrated some of its potential applications by comparing analysis pipelines and variant calling parameters and exploring the effects of differences in coverage. As both sensitivity and specificity are influenced by various experimental factors, including sample preparation, the sequencing itself and the bioinformatic pipelines used to analyse data, the procedure could be used to assess the performance of a sequencing experiment globally and could complement other commonly used approaches, such as the assessment of base call quality or of coverage metrics. The main advantage of the method presented here is that it does not require a reference technique, such as genotyping using microarrays; a reference method in itself may be problematic, as its overall error rates have been reported to be in the order of 10^–^^2^ (Li *et al.*, 2013). However, our method relies on the availability of appropriate allele frequencies.

The use of allele frequencies has two consequences. The first is that compared with the situation where the presence or absence of variants is known, using a probability introduces a degree of uncertainty that is reflected in a larger scatter of the estimates (Table 1). The second is that it forces one to decide which set of allele frequencies to use. The choice of the allele frequencies from a specific population, or, if available, from a particular subpopulation, disregards the possibility that individuals may represent a mixture from different populations. This problem could be circumvented using a more complicated machinery that considers, for example, the probability of belonging to a certain population, or of carrying certain haplotypes. Misspecification of the population frequencies will influence the specificity and sensitivity estimates, but Figure 5 suggests that if two procedures are significantly different in specificity or sensitivity, misspecification of the allele frequencies will tend to preserve the order.

One of the critical issues is the location of the polymorphisms used. Here we chose those included in the regions targeted by the enrichment procedure. However, as the practical interest is to detect mutations likely to cause disease, a natural choice would be to use all the polymorphisms in coding or perhaps in regulatory regions. This would assess the experiment as a whole including the choice of the enrichment procedure. Another question is the choice of the polymorphisms. Initially we used the polymorphisms included in the microarray. These polymorphisms represent a selection based on criteria that probably include the likelihood of being efficiently typed using microarray technology. This will probably result in avoiding certain types of polymorphisms and polymorphisms in certain locations such as regions with extreme base compositions. It is possible that sequencing experiments are accurate in exactly the same regions. This would lead to overestimation of sensitivity and specificity when using microarray genotyping as reference technique. The method proposed here allows for a comparison of different types of polymorphisms and we showed its application to the identification of indels. As expected, sensitivity appears to be lower for indels than for single-nucleotide substitutions. Although allele frequencies for polymorphisms not included in microarrays may not be accurate, our results are consistent with published studies that show that indel detection is still a challenging issue (Albers *et al.*, 2011; Bansal *et al.*, 2011).

As our interest focused on the detection of rare variants, we were able to dichotomize the outcome by scoring at each position whether a variant was present or absent. This leads effortlessly into the determination of specificity and sensitivity. However, more complicated scenarios are possible, such as assessing the calling of each of the three possible genotypes defined by a variant/reference allele system at each position. Here, however, we chose the more simple approach. Studies frequently focus on sensitivity as opposed to specificity (e.g. Pattnaik *et al.*, 2012); however, estimation of specificity is important, as it may help to assess the amount of validation work that is required, that is closely related to false-positive rate. A specificity of 0.99 and a frequency of deviations from the reference sequence in the order of 1 per 1000 sites would be expected to lead, on average, to ∼10 false-positive findings to one true variant position. Therefore, practically useful methods of variant detection benefit from having specificities that are considerably >0.99. Estimating such a parameter accurately will require examining a large number of sites. For example, simply counting false- and true-negative findings, relying, for example, on microarray data would require at least 10 650 invariant positions to establish the difference between a method that has specificity of 0.999 compared with one with a specificity 0.9999 with 80% power. In this article, we primarily relied on sites that are known to be polymorphic; however, we could also include sites for which there are no reported variants and assume that this implies a low minor allele frequency. This would allow to increase the number of sites used and to improve the ability to estimate the specificity.

As our procedure is simple, it would be possible to use it to optimize analysis parameters, by integrating it into, for example, a variant caller so that it maximizes the sensitivity while not allowing specificity to sink below a certain threshold. Such a procedure would benefit from the fact that the order appears to be insensitive to the choice of population. This would allow for an estimation of the amount of validation work required and the likelihood that a change of interest can really be identified and can guide the design of future experiments.

In summary, we have developed an approach to assess the performance of whole-exome or -genome sequencing experiments that requires only the knowledge of the population frequencies of polymorphisms. Such a method could be easily used to assess new/updated analysis procedures and to inform the choice of analysis pipeline, analysis parameters or even of experimental tools.

*Funding*: This work was supported by the British Heart Foundation [Grant reference: FS/10/008/28146]; S.A.T. and M.E.H. were funded by the Wellcome Trust [Grant Number: WT098051].

*Conflict of interest*: none declared.
